# Fluoroscopy-guided biodegradable spacer implantation using local anesthesia: safety and efficacy study in patients with massive rotator cuff tears

**DOI:** 10.1007/s12306-016-0433-0

**Published:** 2016-11-30

**Authors:** E. Gervasi, E. Maman, A. Dekel, E. Cautero

**Affiliations:** 1 Department of Orthopedic Surgery, Latisana Civil Hospital, Via Sabbionera 45, Latisana, 33053 Udine, Italy; 2 Shoulder Unit, Division of Orthopaedic Surgery, Tel Aviv Sourasky Medical Center, Tel Aviv, Israel; 3 Advanced Orthopedic Clinic, Assuta Medical Center, Tel Aviv, Israel

**Keywords:** Massive rotator cuff tears, Local anesthesia, Biodegradable spacer, Fluoroscopy-guided subacromial implantation

## Abstract

**Background:**

The management of massive rotator cuff tears (MRCTs) is challenging and associated with a high failure rates. Studies have shown that advanced age, lower American Society of Anesthesiologists physical status score and concomitant comorbidities are associated with higher risks of death and postoperative complications. This study was designed to assess the safety and efficacy of fluoroscopy-guided biodegradable spacer implantation under local anesthesia, in patients with MRCT and comorbidities completely or partially contraindicating surgeries under general anesthesia.

**Methods:**

In this open-label, single arm, prospective study, subjects with MRCTs underwent subacromial fluoroscopy-guided implantation with a biodegradable spacer (InSpace™ system) under local anesthesia. Fifteen patients were treated and assessed. Follow-up visits were scheduled according to routine clinical practice. Shoulder function was evaluated using Constant (CS) and American Shoulder and Elbow Society (ASES) scores.

**Results:**

All patients demonstrated an overall improvement in the total CS and ASES beginning at 6 weeks and sustained by at least 12 months postoperatively. Of the 15 patients who reached the 1-year follow-up, 85% showed a clinically significant improvement of at least 15 points in their Constant score starting at 6 weeks postoperation and maintained throughout the entire follow-up period.

**Conclusions:**

We conclude that in this initial patient’s cohort, fluoroscopy-guided implantation of InSpace™ system under local anesthesia, represented an effective alternative to the existing procedures. This procedure may be considered as a treatment option for elderly patients or for patients with multiple comorbidities complicating or contraindicating surgery under general anesthesia. Technically easy, this technique can be an effective tool in the armamentarium of most orthopedic surgeons. *Level of proof:* single-arm prospective study, Level II.

## Background

The management of patients with massive rotator cuff tears (MRCT) remains a challenge for orthopedic surgeons. In complex MRCT cases, treatment options are often limited to total shoulder arthroplasty or tendon transfer. To date, only few non-arthroplasty surgical options exist for the treatment of MRCTs. In cases of complex, non-repairable MRCT, the choices are: tendon transfers for active patients and shoulder reverse arthroplasty for the eldest or when arthritic changes involve the joint. Open or arthroscopic debridement of the RCT and acromioplasty may be appropriate for low-demand patients [1–4]. In cases where pain originates from the humeral head, biceps tenotomy is applicable [5]. Several studies have shown mixed results regarding RC augmentation with allografts or extracellular matrix scaffolds [6, 7]. Patients presenting with marked weakness and pain but without glenohumeral arthritis in the setting of IRCT may benefit from a tendon transfer [8, 9]. Nevertheless, there is no current consensus or definitive guidelines concerning the optimal surgical treatment for this devastating condition.

Studies in many orthopedic fields such as hip and spine surgery have shown that advanced age, lower American Society of Anesthesiologists (ASA) physical status score [10], concomitant cardiovascular disease, pulmonary disease and diabetes are associated with higher risks of death and postoperative complications [11, 12].

Vast majority of the patients with MRCT belong to the same age group and might suffer from the similar diseases; thus, one could assume that the relative risk of death and postoperative complications will be as high as in the aforementioned fields. Regional (local) anesthesia is therefore believed to decrease postoperative complications by reducing sympathetic activation and inflammation, by preventing venous stasis, and by avoiding tracheal intubation and positive pressure ventilation [13, 14].

Regional anesthesia for upper limb surgery has several advantages compared with general anesthesia, including better postoperative analgesia, less nausea and vomiting, more hemodynamic stability, fewer side effects and a favorable complications profile [15]. However, the so-called blended anesthesia that includes interscalene nerve block adjunct to the general anesthesia is the common choice for shoulder surgery.

The latest treatment modality suggested for MRCT patients is the InSpace™ system [16, 17]. This device is a biodegradable spacer (balloon shape), which is implanted between the acromion and the humeral head and helps to recenter the humeral head relative to the glenoid. The spacer is made of a copolymer poly (l-lactide-co-ε-caprolactone) that is biodegradable and totally dissipates within 12 months of implantation. The device attempts to restore painless shoulder biomechanics by decreasing subacromial friction and by lowering the humeral head during abduction [17]. The biodegradable spacer may be implanted under local anesthesia. Local anesthesia alone for the fluoroscopy-guided InSpace implantation does not require low systolic blood pressure maintenance as compared to the other arthroscopic procedures.

Patients with chronic progressive RC tears often develop the ability to compensate for their deficient RC without even being aware of this learned behavior. By inserting this biodegradable spacer, the shoulder is enabled to potentially learn this compensatory behavior, thus allowing the patient to develop a chronic compensated and asymptomatic RC tear-type shoulder. During this period, the device permits the humeral head to glide smoothly without friction under the acromion, thus permitting longstanding improvement in glenohumeral joint motion with significant pain reduction.

This study aimed to assess the safety and efficacy of the InSpace implantation under local anesthesia in patients with multiple comorbidities, contraindicating more invasive surgeries, such as reverse arthroplasty, within a period of at least one year following the surgery.

## Patients and methods

The Udine Regional Ethics Committee (Italy) reviewed and approved the study, and each of the participating patients gave their written consent as required prior to any study procedure. In the first cohort of the study, fifteen (15) patients including 8 (53%) females and 7 (47%) males with a mean age 74.6 (SD: 6.5; Median: 23.7) (Table 1) met the inclusion criteria and were enrolled. The criteria for inclusion were age 50 years or older, imaging confirmation of a RCT by MRI and documented failure of conservative therapy. Patients with evidence of significant osteoarthritis, or cartilage damage in the shoulder, significant glenohumeral instability, major joint trauma, infection or necrosis in the shoulder were excluded. Patient demographic information, non-orthopedic comorbidities, type and severity of the shoulder injury, pain level and baseline physical function were recorded.

**Table 1:** Baseline patients’ characteristics

Age	74 ± 6
Gender (female/male)	8/7
Dominant arm involved (yes/no)	11/4
VAS pain scale (0–10)	7.1 ± 1.6
Total ASES (0–100)	24.5 ± 14
Total CS (0–100)	31.9 ± 14
Fatty infiltration (III/IV)^a^	6/9
Previous conservative treatment: steroid injection/pain medication/PT	10/2/3
Prior surgery of rotator cuff (yes/no)	5/10
Time from RCT diagnosis till operation	7 ± 4.6 months

Values are expressed as mean ± standard deviation or in number of patients

^a^Fatty degeneration is classified according to modified Goutallier et al. [21] as diagnosed by baseline MRI

All patients were symptomatic and complained of persistent shoulder pain for a minimum of 4 months. The mean period from first complaint of shoulder pain to operation was 47 months (SD: 60.5, Median: 16.6), while the time from diagnosis up to surgery was 7 months (SD:4.7, Median 6.6) (Table 1).

All patients had previously failed RC syndrome treatments such as steroid injections and physiotherapy. Of these, 5/15 (33%) had been previously treated surgically with either cuff repair or debridement and biceps tenotomy.

The mean preoperative visual analogue scale (VAS) for pain was 7.1, and the mean TCS and ASES were 31.9 and 24.5, respectively.

### Surgical procedure

All procedures were performed by two, fellowship-trained shoulder surgeon (EG and EC). Briefly, implantations were performed utilizing fluoroscopy procedure with the patient seated in either a beach-chair position or lateral decubitus position, under local anesthesia and fluoroscopic guidance as was previously described [18].

The local anesthesia was performed first, by injecting Lidocaine 2% and Marcaine 2% into the subacromial space, at the lateral incision situ and around the anterolateral acromion edge.

**Fig. 1:**
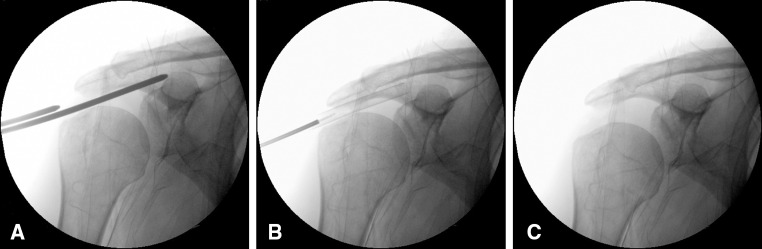
Fluoroscopy-guided implantation of subacromial biodegradable spacer. **a** Subacromial space measurement. **b** Confirmation of device insertion by fluoroscopic view. **c** Confirmation of placement

A lateral incision 1.5 cm long matches the lateral arthroscopic portal. The device implantation proceeds by inserting two identical metal rods, verified under fluoroscopic vision, until it overhangs 1 cm medially the glenoid rim. The proper position was verified using the fluoroscopy (coronal and axial view) (Fig. 1a). The outside part of the rod was measured to indicate the needed spacer size (small, medium or large).

The device implantation was completed by inserting the protecting sheath until it was 1 cm medial to the glenoid rim, while following the direction of the scapular spine (to avoid too anterior or posterior position). Correct position was verified using a lateral fluoroscopy view (Fig. 1b).

At this stage, we preferred to push the protecting sheath over the rod; while the sheath was in place, the rod was retracted and replaced by the rolled spacer (balloon). This “out of the role” maneuver with the rod, that does not bend, allows maintaining the right position during the whole insertion.

Once the position is assured, the device is inflated using physiological solution: at the maximum filling volume first, to spread the spacer, then the volume reduced to the recommended value by withdrawing a definite amount of the solution, as per each device size-specific instructions in the product labeling.

The spacer (balloon) is then sealed and secured in situ; the delivery system removed and the skin closed. The humeral head position and the acromion-humeral distance increase are confirmed by fluoroscopy, determining the accurate placement of the implant (Fig. 1c).

### Outcome measures

The same orthopedic surgeon (E.C.) assessed the pre- and postoperative shoulder function at each visit until the “two years” postimplantation follow-up. Physical examination, ROM and shoulder function were assessed by Constant score (CS) and American Shoulder and Elbow Society (ASES) [19] evaluated postoperatively at the following time points: 2, 6 weeks, 3, 6, 12 and 24 month. Primary outcomes were defined as final total functional scores (Constant and ASES scores).

Ultrasound was done to all patients up to 3 months postimplantation to verify device positioning.

### Statistical analysis

Study data were analyzed with the SAS^®^ version 9.2 (SAS Institute, Cary NC, USA). For comparison of means (continuous variables), the two-sample *t* test or the Wilcoxon rank sum test were used. For comparison of proportions (categorical variables), the Chi-squared test or Fisher’s exact test was as appropriate. The mean changes from baseline in total CS and adjusted CS and its subscales were determined using a repeated measures analysis variance model. *p* values <0.05 were considered statistically significant with no adjustment for multiple testing.

## Results

Fifteen patients were treated and assessed. This patients group demonstrated an overall statistically and clinically significant improvement in the total CS and ASES beginning at 6 weeks and sustained by 24 months postoperatively.

Of the 15 patients that completed a minimum of 1-year follow-up, 80% presented good effectiveness results, including rapid pain relief and restoration of active and painless motion and improvement of at least 10 points in the total Constant score (TCS) starting at 6 weeks postimplantation, which maintained over time. Ten (10) of these patients (66.6%) also completed 2 years of postoperative follow-up in which the improvement in shoulder functionality and pain was sustained.

**Fig. 2:**
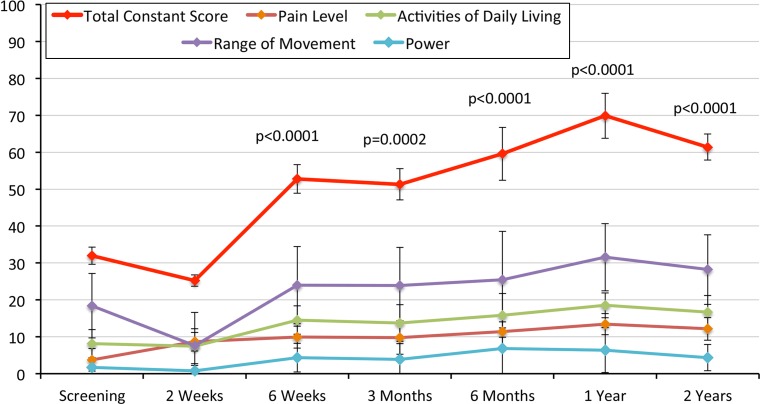
Graphical presentation of Constant variables following biodegradable spacer insertion. Values are presented as mean ± SD

The total CS has improved significantly from a mean of 31.9 point at preoperative (baseline) to 69.8 points at 12-month postoperation and maintained at the similar improvement level for 70% of the ten patients that completed the 24-month follow-up (Fig. 2).

**Fig. 3:**
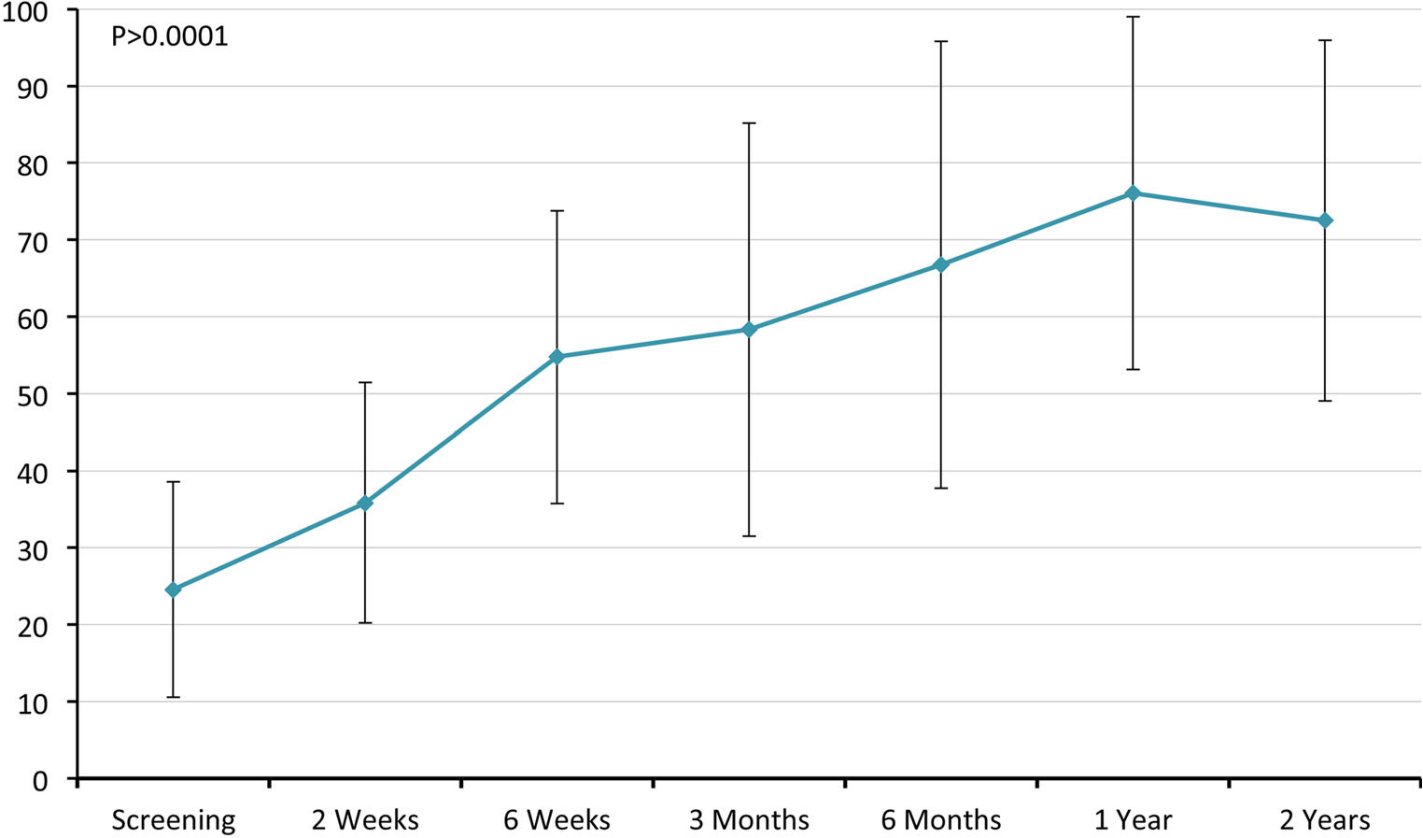
Graphical presentation of total ASES score following biodegradable spacer insertion. Values are presented as mean ± SD

The ASES score has improved significantly (pre-op/post-op (12 m/24 m): pain (VAS) 7.1/1.4/2.1; ADL 6.1/19.3/19.8 TOTAL 24.5/76/72.5) (Fig. 3). Majority of patients (13/15, 80%) were scored their satisfaction of the surgical procedure as 8–10 (in a scale on 0–10, where 10 is very satisfied).

**Fig. 4:**
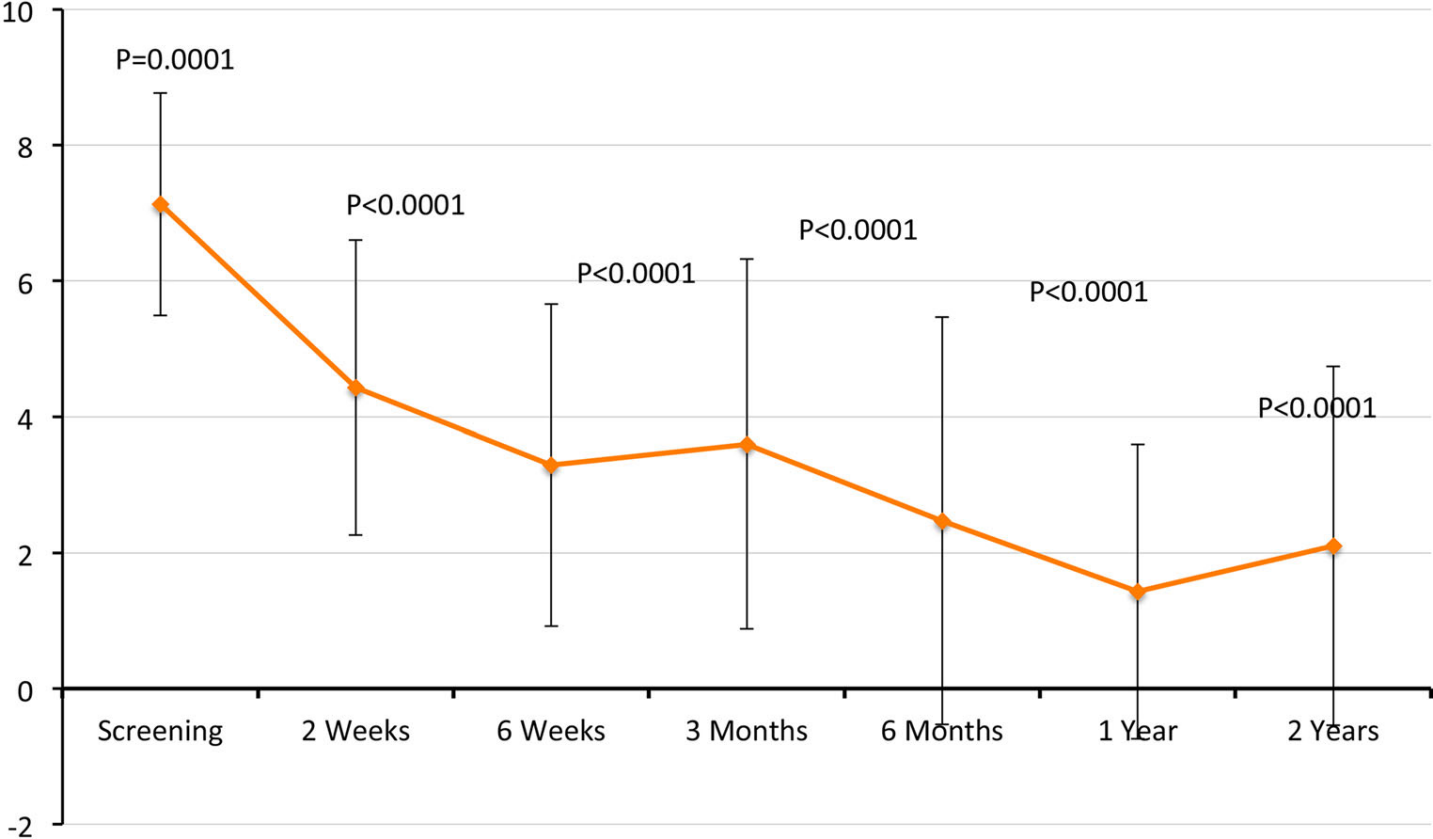
Graphical presentation of VAS obtained from ASES score. Values are presented as mean ± SD

Pain parameters of both CS and ASES (VAS) as well as ROM improved significantly (*p* ≤ 0.005) starting 2-week postimplantation procedure, while other shoulder parameters (ADL, ROM and had power/strength) had improved starting 6-week postimplantation (Figs. 2, 3, 4).

All patients beside two (20%) were discharged at the same day of the surgery and did not complained of any postoperative effect such as nausea and vomiting or hemodynamic instability. The two patients that remained overnight stayed either due to their elderly age that required closer observation or due to the distance from hospital to home that required overnight stay.

Mean implantation time was of the device was approximately 10 min.

The device implantation time ranged from 5 min in 28% of the procedures to 20 min in 5% of the cases when the surgeon faced some technical constrains. Device was rated as very easy for use by the surgeon with a mean score of 8.7 in a scale of 1–10 (where 1 is very difficult and 10 is very easy to deploy and operate).

During the postoperative period, no serious or clinically significant device-related adverse effects were observed. Only one patient (1/25; 0.4%) prematurely discontinued his participation in the study at 6-month postimplantation due to insufficient improvement and was referred to a shoulder arthroplasty.

## Discussion

The principal results of this study demonstrated a significant improvement in the shoulder function in patients with MRCTs following fluoroscopy-guided implantation of the InSpace™ system under local anesthesia. One-year follow-up revealed a significant improvement in the total CS and ASES (including pain scores, nocturnal pain, range of movement and activity of daily living) commencing at the early postoperative stages, and continued with further improvement throughout prolonged follow-up.

This study results are in a line with those reported by Senecovic et al. [15]. Senecovic et al. reported a significant increase in the mean total CS from 33.4 to 65.4 points at 3 years. There was an improvement of 6.4 points in subjective pain score, which commenced at 1 week postoperatively and was sustained until 3 years of follow-up. Improvement in power was only evident at 18 months of follow-up but was sustained at 3 years.

Efficacy results of the current study are comparable with those reported in a series of patients treated with existing techniques. Rockwood et al. [4] reported 83% satisfactory results using debridement, subacromial decompression and acromioplasty of massive degenerative IRCTs with an average follow-up of six years. Arthroscopic repair of massive rotator cuff tears with stage 3 and 4 fatty infiltrations resulted in significant functional improvement as reported by Burkhart et al. [20]. With mean follow-up of 39 month, Burkhart et al. demonstrated a clinical improvement for some patients having >75% fatty degeneration and for all patients in the 50–75% group. Current study suggests a less invasive surgical procedure that can be done in an outpatient clinic using local anesthesia.

Nevertheless, the main limitation of this study is the small number of patients with a relatively short follow-up period. As the initial protocol was intended to observe the outcome of such challenging patients’ population, the study sample size was increased to 45 patients in order to present more powerful and statistically significant efficacy outcome. Also the fact that the study has no comparative arm is a downside; however, the selected patients were failed the common treatment of MRCT and more than 30% of them failed a combination of both surgical intervention and conservative treatment. Hence, we believe that at this specific indication, there is no suitable comparative arm and it is appropriate to use each patient as its own control, the mentioned extension of the study to 45 subjects will overcome this challenge.

*In summary*, the data strongly suggest that fluoroscopy-guided InSpace implantation under local anesthesia is a low-risk, clinically effective option, especially for the elderly population and those patients suffering for multiple comorbidities or with contraindication to general anesthesia. It can be alternative to the reverse prosthesis or to tendon transfers in patients with MRCTs without arthropathy.

The procedure of insertion of the InSpace under local anesthesia can be carried out in a day-care or outpatient setting; it is technically easy. This last aspect gives a chance to patients living in areas where the shoulder surgery is still developing.

 Since currently there are neither consensus nor guidelines for the best surgical option in this MRCTs challenging patient population, we conclude that fluoroscopy-guided InSpace implantation is an effective alternative to the existing procedures, arthroscopic or open, for patients having painful massive rotator cuff tears refractory to surgical or conservative managements. A controlled trial with larger cohort of subject for longer follow-up period for the clinical and functional outcomes following fluoroscopy-guided InSpace ™ implantation will further establish the results and outcome of this initial study cohort.
